# Social-Ecological Changes in a *Quilombola* Community in the Atlantic Forest of Southeastern Brazil

**DOI:** 10.1007/s10745-014-9691-3

**Published:** 2014-08-23

**Authors:** Kjersti Thorkildsen

**Affiliations:** Department of International Environment and Development Studies, Noragric, Norwegian University of Life Sciences, P.O. Box 5003, NO-1432 Ås, Norway

**Keywords:** *Quilombola*, Shifting cultivation, Atlantic Forest, Biodiversity conservation, Brazil

## Abstract

Through a combined adaptive cycle and political ecology approach, this article explores how the Afro-Brazilian *Quilombolas* of Bombas, living inside the protected area of PETAR, respond to and shape social-ecological changes in the Atlantic Forest. Field data reveal that both environmental restrictions and social policies of state transfer payments and food packages have contributed to decreased engagement in agricultural practices, loss of traditional knowledge, and reduced agro-biodiversity. The claim to land rights based on a *Quilombola* identity and recent negotiations with forest authorities insinuate a shift of this trend. Contrary to dominant conservation narratives, the findings indicate that small-scale shifting cultivation practices by the *Quilombolas* have the potential to increase structural ecological complexity of the Atlantic Forest. The article therefore argues that legalization of settlement and subsistence activities is important not only for livelihood security and social cohesion of Bombas inhabitants, but also possibly for biodiversity conservation.

## Introduction

The issue of *Quilombos* 1 entered the Brazilian political scene with the promulgation of the renewed and more democratic Federal Constitution of 1988 following the end of military rule (1964–1985). With article 68 of the Temporary Constitutional Provisions Act, remnants of rural Afro-Brazilian *Quilombola* communities were for the first time recognized as rightful owners of the land they occupied (Rapoport Center 2008). Several *Quilombola* communities are situated in the Ribeira Valley in the State of São Paulo in southeastern Brazil, linked to the early historical introduction of slaves for use in gold mining in the sixteenth century (Queiroz 1983; Oliveira Jr *et al.* 2000). With the demise of mineral extraction in the beginning of the eighteenth century, the Ribeira Valley became a region where slaves were freed or abandoned earlier than in other parts of the country^2^ (Castro *et al.* 2006; Diegues 2007). According to the Coordination and advisory team for black and *Quilombola* communities in the Ribeira Valley (EEACONE), 88 *Quilombola* communities live in the region (Andrade and Tatto 2013). Of these, Bombas is often considered to be the most remote and traditional, but has not yet been officially recognized (Santos and Tatto 2008; Santos 2010). The Land Institute of São Paulo’s Technical and Scientific Report, based on an anthropological study, points to strong community ties and characteristics of a *Quilombo*, concluding that Bombas adequately fits the legal criteria for due recognition (Silveira 2003). However, as Bombas is situated inside the Upper Ribeira State Touristic Park (PETAR), the recognition process was halted by São Paulo Environmental Office in 2003, demanding environmental studies of the territory. Because of the historically strained relationship with forest authorities, Bombas inhabitants refused the entrance of researchers to undertake such studies before being recognized. Meanwhile, as the process remains deadlocked, community residents are excluded from access to social services and infrastructure development.

This predicament derives from Brazil’s adoption of the North American “fortress approach” to conservation in the 1930s, where human occupation and resource extraction were deemed incompatible in protected areas^3^ (Diegues 1998; Penna-Firme 2013). PETAR was the first protected area to be established in the State of São Paulo in 1958 and was based on a notion of “wilderness” without human interference. The prime goal was to protect more than 350 limestone caves from mining, “virgin” Atlantic Forest from logging, and endemic fauna and flora from extraction (Fundação Florestal 2010). When the boundaries of PETAR were drawn, the entire territory of Bombas was included in the more than 35,000 ha Nature Park. No consideration was given to the inhabitants of Bombas; rather, their subsistence practices and residence became prohibited by law (Silveira 2001). However, it was not until 1987 that efforts were made to implement decree 32.283/1958 establishing PETAR, and community members began to face environmental restrictions on their resource use and threats of eviction.

The majority of protected areas created during Brazil’s military dictatorship persisted as “paper parks” until the mid-1980s when international and national pressure from conservation organizations triggered implementation, ultimately leading to violations of land rights and social marginalization of expelled forest inhabitants (Diegues 2011). Since then, because of disruption of residents’ livelihoods and poor environmental protection results, this “fortress approach” to conservation has been criticized by social-environmental movements and organizations, social scientists, and more recently, a growing number of natural scientists worldwide (e.g., Gomez-Pompa and Kaus 1992; Stevens and de Lacy 1997; Neumann 2004; Brockington *et al.* 2008; Oudenhoven *et al.* 2011; Robbins 2012; Benjaminsen and Bryceson 2012). A number of scholars have questioned equilibrium theories of climax forests in stable states, often used in support of creating strictly protected areas aimed at reducing variability by applying external controls (e.g., Fairhead and Leach 2000; Zimmerer 2000; Forsyth and Walker 2008; Beymer-Farris 2013). These critics emphasize the importance of small-scale disturbances caused by human actors in producing biologically diverse forests in multiple states. Moreover, in Brazil, the significance of traditional peoples’ knowledge and their balanced relationship with the Atlantic Forest has been raised and used as an argument to legalize their settlements within these areas (Sanches 2001; Ferreira 2004; Rezende da Silva 2008; Diegues 2011).

To explain how the *Quilombolas* of Bombas respond to and shape social-ecological changes, this article explores historical and contemporary social, ecological, economic, and political processes that have affected their livelihoods and the Atlantic Forest. Even though the majority of *Quilombos* in the Ribeira Valley are situated in forest areas, most studies of such communities have either looked at social or ecological aspects, treating these dimensions separately. Few studies have analyzed the way *Quilombolas*’ cultural dynamics and subsistence strategies have changed over time and how this has shaped and maintained the Atlantic Forest, and even fewer studies have taken into account the political dimensions of these changes (Pedroso *et al.* 2008; Pedroso *et al.* 2009; Munari 2009; Adams *et al.* 2013). I seek to investigate these gaps by adopting an interdisciplinary approach.

## Theoretical and Methodological Approach

As a means to analyze processes of change in the social-ecological system of Bombas, I combine the adaptive cycle much used in resilience literature with insights from political ecology. The adaptive cycle was originally developed by Crawford Stanley Holling (1986) who also introduced the concept of ecological resilience in an effort to study how ecosystems cope with and adapt to change at various spatial and temporal scales. In contrast to stable equilibrium assumptions, Holling’s research highlights ecosystems’ multi-equilibrium dynamics and cyclical nature. According to the adaptive cycle, an ecosystem proceeds from fast growth (exploitation - r) slowly to a climax community (conservation - K), then rapidly to collapse or release (creative destruction - Ω), and rapidly to reorganization (renewal - α), before returning to the growth phase (Holling 1986). During the long, slow progression from r to K, organization or connectedness is increased accompanied by gradual accumulation of capital. As stability increases, variability and diversity decreases and there is a diminished likelihood that novelty will arise. The ecosystem eventually becomes so over-connected that rapid discontinuous change is triggered leading to stored capital being released, which may result in some attributes of the system being lost. This is followed by a period of reorganization during which innovation and adaptation can take place. In the following r phase, the system settles into a new trajectory.

The concept of the adaptive cycle has in more recent years been further developed to analyze integrated social-ecological systems and adaptive management (e.g., Gunderson and Holling 2002; Seixas and Berkes 2003; Widlock *et al.* 2012). The social science component is, however, still relatively weakly developed and society is often portrayed as a closed system devoid of human agency. Moreover, the “social-ecological resilience” approach, upon which the adaptive cycle builds, has been criticized for being ahistorical and for not sufficiently addressing social justice, power relationships, and the role of politics in shaping resource access and control (Turner 2008; Davidson 2010; Beymer-Farris *et al.* 2012; Beymer-Farris 2013). As a way to expand the theory of the adaptive cycle to also incorporate historical and political dynamics and human agency, I have chosen to integrate it with insights from political ecology inspired by Beymer-Farris (2013). The field of political ecology places emphasis on how institutional history and existing political-economic structures and embedded power relations influence resource access and management, and has been employed to examine political struggles and adaptive capacities of human societies (e.g., Fairhead and Leach 2000; Zimmerer 2000; Neumann 2004; Porro 2005; Robbins 2012). Political ecology offers a critical perspective on biodiversity conservation and the generally problematic relationship between protected areas and human communities.

### Methods

The primary data used in this article were obtained from ethnographic fieldwork in Bombas with participant observation and registration in a field diary, as well as 30 recorded open- ended in-depth interviews with community members, former inhabitants, leaders from other *Quilombola* communities in the municipalities of Eldorado and Iporanga, government officials, politicians, lawyers, researchers, teachers, tourist guides, and representatives from NGOs, social movements, and religious orders (2010–2013). Insight was also gained from informal conversations, attending meetings, public hearings, and seminars with community members and other key actors. Historical data were obtained from traditional oral accounts and combined with official documents and publications, contributing towards the reconstruction of the social, economic, and political past of the Ribeira Valley. Additionally, changes in land cover and forest patterns in Bombas through time were analyzed by classifying and comparing an aerial photo from 1962 and three satellite images from 1990, 1999, and 2010 with ArcGis software^4^. Land cover was classified into three categories: (1) agricultural activities: home gardens, cultivation plots, and recent fallows of up to 3 years; (2) regenerating forests of 4–10 years; and (3) forest areas > 10 years, calculating the size and number of patches in each class. We should be mindful of the fact that the resolutions of the satellite images were not equal, namely, 30 m for the Landsat image of 1990, 15 m for the Landsat image of 1999, and 2.5 m for the SPOT image of 2010. This may have affected the visual assessment of land cover. Classification of land use in the four periods and interpretation of the observed changes were therefore cross-checked with the inhabitants of Bombas in a focus group discussion held in the community in April 2013.

## The Community of Bombas

**Fig. 1:**
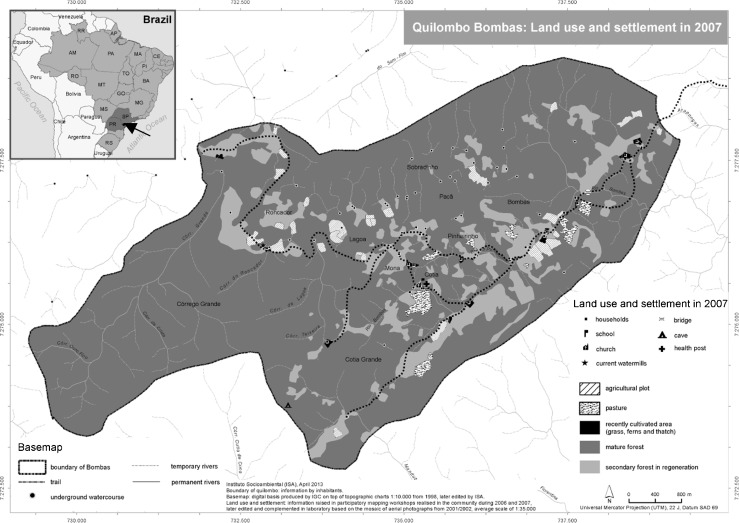
Map of the territory of Bombas showing land use and settlements in 2007 (Santos and Tatto 2008)

Bombas is located in the municipality of Iporanga, about five kilometers from the dirt road linking Iporanga and Apiaí. Because of its steep terrain, access to the community is difficult and time-consuming. The only way to reach Bombas is by foot or on horseback. Historical use and occupation have given rise to a territory of 3,229 ha (Fig. 1). All areas in Bombas have been inhabited, but Córrego Grande has been left fallow for many years. The landscape is characterized by a mosaic of mature forest, secondary forest in regeneration, and recently cultivated areas. The bedrock is principally limestone with many underground caves (Fundação Florestal 2010). Twenty-seven houses made of wood and clay are scattered throughout the territory and there is no village center (Santos and Tatto 2008). However, inhabitants refer to two nuclei as Bombas and Cotia where the two schools are situated. The majority of inhabitants are illiterate and education services are meager, offering classes from only the first to fourth grade in elementary education. In addition to having no road access, there is no electricity, basic sanitation, garbage collection, health services, or public phone in contrast to other *Quilombola* communities in the Ribeira Valley. The community center is in Bombas, while the once-important chapel, now in ruins, is located in Cotia.

### The History of the Bombas Settlement

According to interviewees, the Bombas valley used to be crossed by indigenous peoples who migrated southeast from the high plains in search of fish and mollusks on the Atlantic coastline. Archaeological studies carried out by de Blasis and Robrahn (1998) support this claim, showing that the valley was a pre-historic route of communication between the Atlantic highland and the lowland of Ribeira. In Bombas, arrow heads can be found in many archaeological sites and inhabitants talk about an indigenous cemetery in Cotia (Silveira 2003). Indigenous peoples have had a vital role in the Ribeira Valley in giving names to geographic locations, fauna, and flora as well as inventing tools for hunting, fishing, and agriculture (Diegues 2007). The practice of shifting cultivation is an indigenous heritage representing adaptations to household mobility and subsistence economy (Candido 1964). Manioc cultivation and the processing of flour is a practice adapted to soil and rainforest conditions, also originating from indigenous peoples (Adams *et al.* 2013). Even though Bombas has been used and occupied sporadically for hundreds or thousands of years, no titles were registered in the area until 1855/56 when 16 persons claimed to possess land (Silveira 2003). However, this does not necessarily imply that these people lived there and used the land. Ângela Ursulino de Freitas from Baú is considered one of the first inhabitants with kinship relations to contemporary inhabitants, settling around 1910. According to her grandchildren, she used to be a slave. What is apparent from Silveira’s study (2003) and also from Bombas inhabitants’ statements is that the origin of the community stems from different occupations of people with varied backgrounds. Despite this, the community members see themselves as one group united by kinship, affinity, and work.

### Traditional Resource use in the Atlantic Forest

At the time of the settlement of Bombas, natural resources and land were abundant and it was possible to freely choose areas for building of houses and opening of agricultural plots. An agricultural plot belonged to the person who first cleared and farmed it, and according to the degree of kinship, this “owner” could assign planting rights to relatives. Through farming, the community members of Bombas could ensure calories and protein in their diet, growing annual and perennial food crops. Small home gardens were planted with a wide variety of vegetables, herbs, and fruits and short-term subsistence crops such as rice, beans, maize, sugar cane, and manioc were cultivated in agricultural plots using shifting cultivation techniques. This agricultural system is widespread throughout tropical forest habitats in Brazil (Sanches 2001; Porro 2005; Pedroso Jr. *et al.* 2009; Hanazaki *et al.* 2013) and in other tropical areas in the world (van Vliet *et al.* 2012). Physical conditions, such as forest age (stage of succession), soil properties, and historical use were considered when a plot was chosen for cultivation. The steepest and rockiest areas were generally avoided and therefore covered by mature forest. A forest patch in secondary regrowth was preferred as it was rich in organic matter and trunk diameter was low and therefore less labor intensive to clear. Undergrowth was first removed by hoe followed by the cutting of trees with an axe. After being left to dry in the sun, the area was burnt. Experienced elders decided when to set fire and monitored the plot to control the spread of fire. According to Forsyth and Walker (2008), controlled burning practices can systematically enrich both forest and fallow vegetation as stored nutrients are released and added to the soil resulting in increased biomass production, while fire also stimulates seed dispersal and controls disease and pest outbreaks. After being cultivated for a couple of years, fields were fallowed for a considerable time (5–30 years) before replanting or left to completely regenerate. It was not uncommon for a family to have many plots, some located far away from the home compound. Some plots were more intensively used, such as those in closer proximity to homes, and some were abandoned for longer periods, creating a heterogeneous land cover composed of a complex mosaic of cultivated areas, primary forest, and secondary forest.

All agricultural activities were assigned their respective months and timed with moon phases in order to achieve optimal output (Sanches 2001). Rice was generally planted at the end of the dry season in November and harvested in May by a collective effort (*puxirão*)^5^ involving community members as well as relatives and friends from nearby areas (Silveira 2003). Beans were often planted together with maize, helping to fix nitrogen, and could be planted two or three times a year depending on the climatic conditions. As cultivated and wild varieties of manioc were planted close to each other in the small agricultural plots opened inside the natural vegetation, gene flow was maintained through hybridization contributing to augment diversity (McKey *et al.* 2010). Bitter varieties of manioc were generally preferred because of pest resistance and as they were not generally eaten by most animals, but they had to be processed into flour (Adams *et al.* 2013; Hanazaki *et al.* 2013). Sweet manioc plots were invaded particularly by ungulates such as collared peccaries, white-lipped peccaries, and deer (Prado *et al.* 2013), as well as rodents such as paca and agouti. Other cultivated fields and home gardens were also often visited by these animals in addition to lowland tapirs, armadillos, and a wide variety of birds. Hunting of such animals, typically a male activity, involving the use of rifles, traps, and dogs, was mainly practiced either to complement the diet or to protect home gardens and cultivated fields (Prado *et al.* 2013).

Mature forests were sporadically utilized to obtain hardwood for house construction and vines to make handicrafts and utilitarian objects such as baskets and sieves, and for roof thatching. Secondary vegetation close to the houses was used as firewood. It was generally a woman’s task to collect forest products, including medicinal plants, and to plant home gardens, while men cut trees and worked in the agricultural fields. Both men and women were involved in processing of manioc and maize flour and production of sugar cane sweets. The inhabitants were mainly self-sustained and conducted little exchange of agricultural products with outsiders. If they produced excess food, they would sell crops and processed flour in Iporanga or Apiaí where there were storage facilities. The local markets provided other daily necessities such as kerosene and salt.

## Pressures and Responses

The relatively stable way of life described above changed in response to four events: (1) the increased investment in the region in the 1930s–1970s, (2) the implementation of PETAR during the 1980s–1990s, (3) the process of construction of a *Quilombola* identity and access to social programs in the 2000s, and (4) the negotiation over land rights with forest authorities from 2010 to 2013. Below, I outline the chronology of changes in social organization, traditional practices, and resource use in Bombas, showing that economic and political incentives have greatly influenced community dynamics and subsistence activities.

### Increased Investment in the Region in the1930s–1970s

After more than a century of economic stagnation in the Ribeira Valley, exploration of mineral deposits was presented as a remedy to the region’s “backwardness,” and governmental investment was initiated at the end of the 1930s. The first important investment was the opening of the Lead and Silver Company in Apiaí and the mining companies Furnas and Lageado in close proximity to Bombas (Silveira 2003). The lack of roads made Iporanga isolated and mining activities difficult and costly, spurring the construction of a road between Iporanga and Apiaí in 1937. Road access facilitated entry of large cattle farmers and the opening of a factory for processing of *juçara* palm hearts (*Euterpe edulis* Mart.) (Figueiredo 2000). Bombas’ population grew as the additional economic alternatives attracted outside workers and their relatives. Many inhabitants turned to the extraction of palm hearts as a main source of income, but household-level agriculture continued to be their main activity for subsistence (Silveira 2001).

Development projects were promoted further in the 1960s in an attempt to occupy abandoned space to counteract rebellions such as the Lamarca guerrilla group^6^, which was present in the Ribeira Valley between 1968 and 1971. A series of infrastructure projects were initiated such as the construction of state road SP-165 linking Iporanga to the municipality of Eldorado, the construction of a bridge over the Ribeira de Iguape River, the provision of electricity and telephone services in Iporanga, and the establishment of a number of regional agencies linked with the political development of the State (Figueiredo 2000). These government projects attracted more people to the region and also to Bombas. According to Bombas inhabitants, more than 80 families used to live in Bombas in the 1970s, resulting in a large extension of cultivated areas. Agricultural plots were sizeable and could have many owners or only one owner who paid a daily fee for help in the field (*camarada*). Despite the intense cultivation, the length of fallow did not change. Community members remember that there used to be a great richness and abundance of animals and birds present in the territory at this time, explaining this bounty by highlighting the extensive availability of crops and fruits. Pig rearing was the main income generating activity, but some inhabitants also raised goats, providing milk and cheese. Others tried for a short while to raise cattle but ceased due to problems of soil compaction and pasture recovery. With increased income, inhabitants could buy cooking oil and dried meat in addition to salt and kerosene.

Collective work was regular and as alliances were comprehensive, it was not uncommon for more than 80 people to participate in *mutirões*, including friends and relatives from nearby communities and towns. *Mutirões* were usually held at the end of every month and a party was organized by the owner of the land plot at the end of the day with large bonfires, accordion and guitar music and dancing and singing until dawn. Elders relate that a domestic animal was slaughtered for the occasion, and manioc flour pancakes (biju) and the local sugarcane spirit (cachaça) were served. The parties were also opportunities for romantic encounters that later resulted in marriage. Other social activities included Catholic masses and celebrations, and priests would visit the community once a month. The celebrations of Bandeira do Divino, Nossa Senhora Aparecida, Santo Antônio, Recommendação das Almas, and the practices of Romario de São Gonçalo and Mesada dos Anjos served to unite the community and reinforce social bonds between community members (Andrade and Tatto 2013).

### Implementation of PETAR in the 1980s–1990s

As a reaction to the extractive activities taking place in Iporanga between the 1930s and 1970s, environmental conservation became a serious concern. Since the Ribeira Valley holds the largest remaining fragment of Brazil’s Atlantic Forest, consisting of 2.1 million hectares, international and national environmental organizations regard Ribeira as a source of natural richness in biodiversity (Ferreira 2004; Santos and Tatto 2008). Furthermore, members of the Brazilian Society of Speleologists and technicians from Brazil’s Geographical and Geological Institute discovered numerous caves that they wanted to preserve in Iporanga and Apiaí, including areas inside and adjacent to the territory of Bombas (Guimarães and LeBret 1966). Among these was the Bombas cave, home to the threatened endemic blind catfish species *Pimelodella kronei* which is one of the environmentalists’ main targets for conservation - and the official logo of PETAR. Based on the speleologists’ findings and suggestions from the Superintendence of Coastal São Paulo, a large number of protected areas was created and implemented in the Ribeira Valley, where PETAR served as a pilot project. In this period, about 70 % of Iporanga municipality was under some form of environmental protection (Figueiredo 2000; Castro *et al.* 2006). Because of conservation policies, palm heart factories were shut down in the mid–1980s and ecotourism targeting urban tourists became the principal focus of governmental actions, with little input from local inhabitants and municipal authorities. Although tourism was developed in the nearby settlement of Serra, it was not in Bombas due to the inaccessibility of this community (Silveira 2007).

News about the implementation of PETAR arrived in Bombas in a confusing way and perplexed its residents. No government official or park staff ever visited the community to inform inhabitants about the creation and implementation of the park. With new park regulations in place, the practice of shifting cultivation and its associated use of fire, planting of home gardens, animal husbandry, hunting, fishing, extraction of palm hearts and other forest products, and human occupation, all became illegal (Andrade and Tatto 2013). Park authorities and environmental police started to appear in the territory to enforce environmental laws, threatening the inhabitants with eviction and charging fines. Inhabitants were sometimes arrested and handcuffed and some started accusing other inhabitants of being involved in illegal extraction of forest resources. This increased tension among community members, which led to a higher incidence of internal conflicts. However, because of its remote location, Bombas was not the target of rigorous monitoring and most surveillance took place near the Bombas cave. Although few Bombas residents were fined for environmental crimes, the fear of the “environment,” as the inhabitants refer to forest authorities, became entrenched in the community (Silveira 2001). Community residents started to suspect any new outsider that came to the area, with fear of having their agricultural plots reported and their rifles confiscated.

The implementation of PETAR left Bombas inhabitants in a confused situation and they were hesitant to engage in traditional agricultural activities. The practice of large collective work efforts like the *mutirão* was avoided in order to not draw attention from park guards and environmental police. However, as Bombas inhabitants had no other options, they continued most resource use practices in more hidden areas with lower visibility and where access of forest authorities became more difficult. This meant that agricultural fields were opened further away from trails and houses and in steeper areas that had previously been avoided. Some steep areas in Bombas are still dominated by ferns, evidence that unsuitable areas were cultivated and have not yet recovered. The situation worsened when some Bombas inhabitants were contracted by external social actors to extract palm hearts. Without other income opportunities, and with abundant populations of *juçara* palms in the territory, the extraction was a way for the inhabitants to make a living. Also, many people from the outside entered the territory to extract palm hearts and young *juçara* palms started to be cut before reaching a reproductive stage, which takes 10 years (Silveira 2001). According to Silveira (2003), this continued until the mid–1990s by which time adult *juçara* palms were almost depleted.

Even though inhabitants were not physically removed from the territory, the threats of forced resettlement and the lack of economic opportunities in the community resulted in many leaving in search of a better life. A large number of inhabitants emigrated to work in tomato plantations in the Upper Ribeira and Sorocaba. Some went to work in sugar cane, pine or eucalyptus plantations, others moved to urban areas of the municipalities of Iporanga, Apiaí, or Itaoca or even further away to Guaraí, Cajaíba, Itu, São Paulo, and Campinas. The inhabitants who did not move, or those who returned because of inadequate living conditions in the outside world, faced new challenges. Few people were left in the community and the low population number made networks of mutual help difficult, resulting in *reunidas* becoming more frequent than *mutirões*. *Reunidas* could be organized any day of the week and involved fewer people and no party at the end of the day. The number and size of agricultural plots and crop rotation decreased, increasing the fallow period. Many elders left and, over time, died resulting in the loss of traditional knowledge about animals and plants, resource utilization and taboos, and making of sweets. The traditional technologies for processing manioc and maize flour also came to an end, leading to the abandonment of the bitter varieties of manioc. The reduced engagement in traditional agricultural activities increased the need for purchasing goods previously produced by local inhabitants such as coffee, soap, sugar cane sweets, and manioc and maize flour. Bombas residents began to depend more on these items, but their purchasing power remained low.

### Construction of a *Quilombola* Identity and Access to Social Programs in the 2000s

With time, Bombas inhabitants came to understand that if they continued hiding their natural resource use practices, they would not manage to cover their subsistence needs and the community would cease to exist. New efforts to plant more were therefore initiated and the number of agricultural plots increased. Simultaneously, the inhabitants started another strategy: to claim territorial rights based on their ethnic identity as *Quilombola*. Living in one of the most isolated areas in the region, Bombas’ inhabitants had been marginal to the discussions about *Quilombos*’ land rights that had taken place since the 1990s in other Afro- descendant communities in the Valley (Silveira 2007). The communities along the Ribeira de Iguape River initiated recognition processes in the beginning of the 1990s as a strategy against the construction of a series of planned hydropower dams. The mobilization of *Quilombola* communities culminated in the establishment of a social-environmental movement of people threatened by dams (Movimento dos ameaçados por barragens - MOAB) with support from the Catholic Church (Commisão Pastoral da Terra). Bombas inhabitants strongly engaged with the Catholic Church were the first to raise the issue of *Quilombo*. Residents began to understand that if the community was recognized as *Quilombo*, their historical territory could cease to be encroached by a protected area, or alternatively be reclassified into a sustainable use area permitting human residence and activity. Park borders had already been adjusted in a number of other *Quilombola* communities in the region such as Ivaporunduva, São Pedro, Maria Rosa, Pilões, and Pedro Cubas, which had been partially inserted in the Intervales State Park (Oliveira Jr *et al.* 2000). These were later reclassified as sustainable use areas, making them part of the Mosaic of Jacupiranga. In 2002, the community of Bombas entered a request for recognition as *Quilombo* to the Land Institute of São Paulo, hoping not only for recognition, but also for the withdrawal of the Park and effective action from the State to improve their living conditions. The community formally organized and registered the Association of Remnants of the *Quilombo* Bombas in 2004.

After becoming socio-politically organized, Bombas inhabitants began to acquire key documents such as birth certificates and identity cards, permitting access to already established governmental social programs. Disabled and elderly people began to receive disability and retirement pensions and started to financially support their families, changing social relations in Bombas. During the Labor Party administration (2003–2013), various programs to fight poverty, hunger, and food insecurity were implemented in Brazil. Bombas residents with children started to receive family allowances (bolsa familia) if sending their children to school. In 2004, the government started to distribute food packages (cesta básica) to Bombas residents, containing items such as rice, beans, maize, flour, sugar, coffee, pasta, and cooking oil, many items traditionally cultivated and processed in the community, thus discouraging engagement in traditional agricultural practices. Fewer agricultural plots were cleared and were frequently situated closer to houses due to time restrictions, given the reduced labor force. Bombas residents no longer planted as much rice, manioc, and other crops as they used to, leading to the abandonment of some varieties and reduced agro-biodiversity. Large-scale collective work arrangements such as *mutirões* and *reunidas* became rare and day-labor exchange (*troca de dia*) came to be the most common form of reciprocal help. Religious celebrations remained the main gathering events in the community (Santos 2010). However, an increasing number of people converted from Catholicism to Pentecostal Evangelical sects, decreasing participation in Catholic celebrations and further weakening social cohesion.

### Negotiation Over Land Rights in 2010–2013

After the completion of the Technical and Scientific Report by the Land Institute of São Paulo (Silveira 2003), based on an anthropological study, the *Quilombola* recognition process was halted by São Paulo Environmental Office which demanded environmental studies of Bombas. The Forest Foundation was charged with conducting these studies, but due to their historically poor relationship with forest authorities, Bombas inhabitants denied researchers entry to the territory until they had been recognized as a *Quilombo*. According to forest authorities, recognition could only be given after environmental studies had been conducted. This deadlock lasted until PETAR started preparing its Management Plan, which involved studying the entire Park, including Bombas. The socio-environmental NGO Instituto Socioambiental (ISA) entered as a mediator in the negotiations between the *Quilombola* association of Bombas, the Forest Foundation, and the Land Institute of São Paulo, resulting in the signing of a Memorandum of Intention and Work Plan in 2010. *Quilombola* leaders from other communities and Catholic Sisters engaged in EEACONE, the legal formalized entity of the anti-dam movement, supported Bombas by sharing experiences and giving legal advice. The Forest Foundation contracted a research group from the Agricultural University of São Paulo (ESALQ) to carry out the studies. After concluding the research report, a proposal of territory was presented by the forest authorities, excluding the area of Córrego Grande. At the time of writing, the Bombas *Quilombola* association had decided to accept this proposal on the conditions that it would be legally recognized, that PETAR’s boundary overlapping Bombas territory would be moved, and that road access would be provided by the State.

At the beginning of this study in 2010, *camaradas* were the most common form of labor organization, whereby a resident remunerates another at the rate of US$ 12 per person per day if s/he encounters difficulties in reciprocating help. This is rather expensive for unsalaried peasants so only individuals receiving retirement or disability allowances could afford it. Twelve families lived in the community at this point, but by 2012 17 families were resident, showing a positive population trend. In April 2013, new plank houses had been built for family members planning to return to Bombas and some residents had replaced their wood and clay houses with plank houses. More time and effort were devoted to community projects, such as the clearing of a soccer field and there was discussion of similarly cleaning the paths. These activities encouraged the return of former residents. A new arrangement of shared agricultural plots was instituted, dividing the work load and the harvest. In this way, social bonds were strengthened and fewer areas needed to be cleared. At this stage residents kept only chickens, ducks, and turkeys rather than pigs, goats, or cows.

## Dynamic social-Ecological Changes in Bombas

### Land Cover Changes

**Fig. 2:**
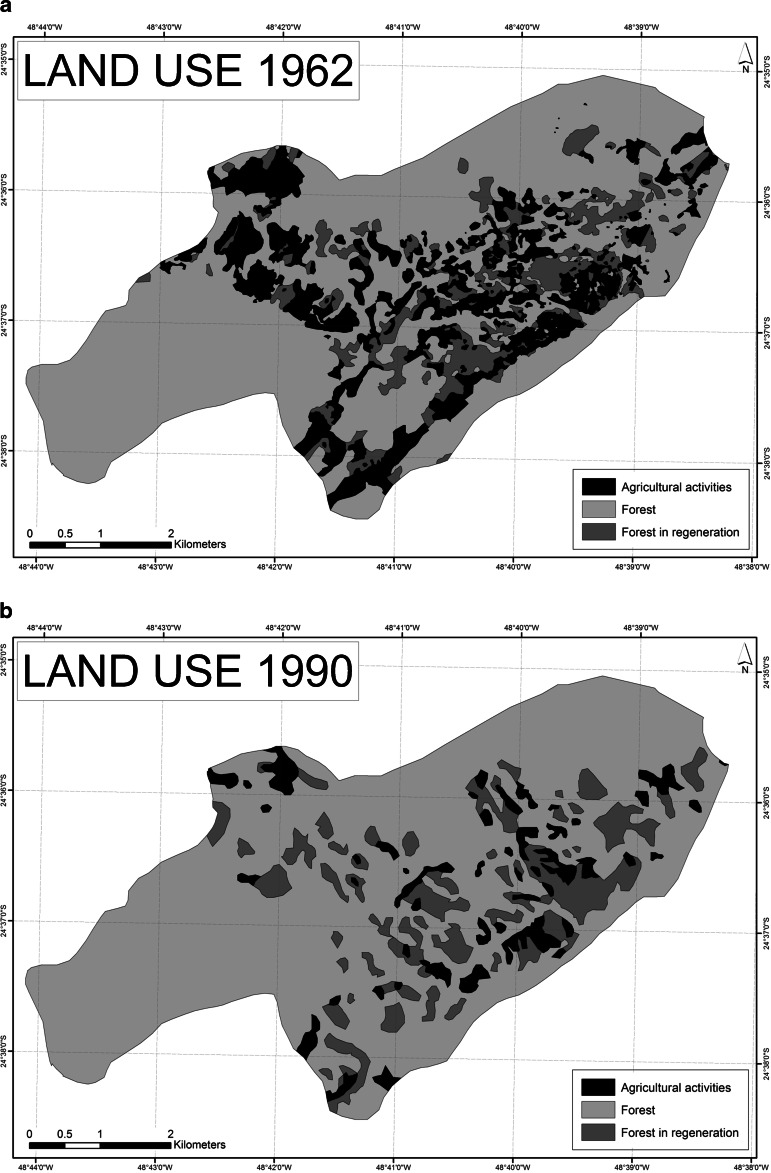

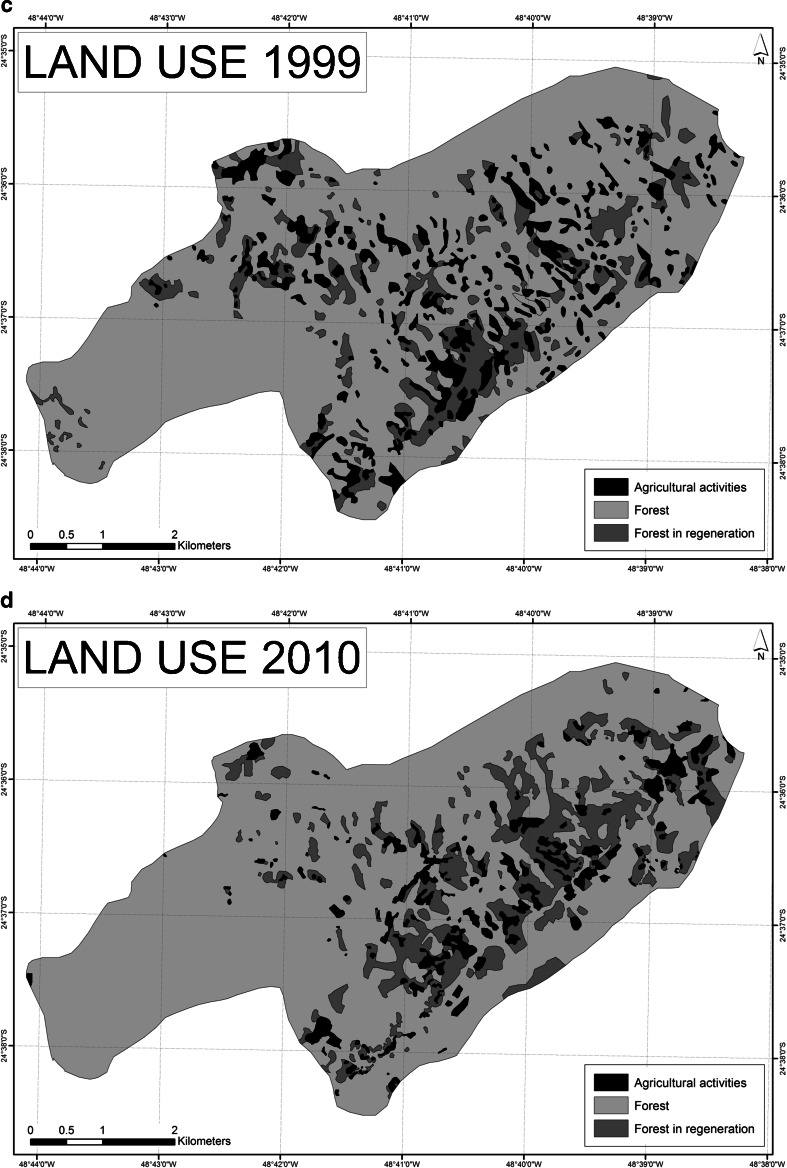
**a**–**d**: Land cover in the Bombas territory showing areas under cultivation, areas in regeneration, and forested areas in four time periods (1962**a**, 1990**b**, 1999**c**, and 2010**d**)

**Fig. 3:**
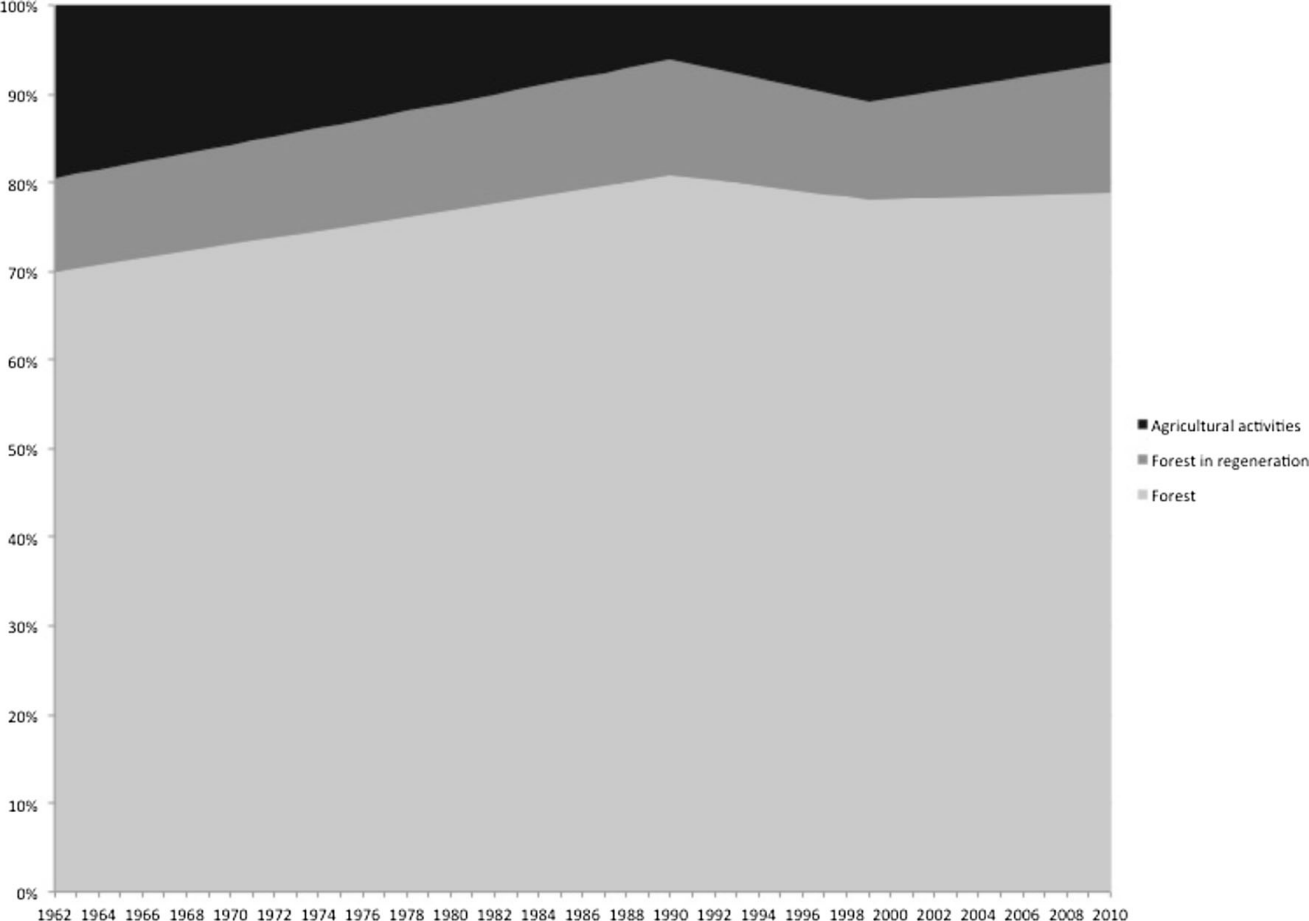
Percentage of the different land cover categories in Bombas over time

Historical changes in land use and forest patterns in Bombas were analyzed by classifying and comparing land cover of an aerial photo from 1962 with satellite images from 1990, 1999, and 2010 (Table 1, Fig. 2a–d). Despite a considerable increase in 1999, which fell again in 2010, the average size of agricultural plots (home gardens, cultivated areas, and fallow) steadily decreased throughout these periods. Residents explained the large size and number of agricultural plots in 1962 (Fig. 2a) as a consequence of high population density and the active involvement in agricultural activities with extensive collective work. This changed after the implementation of PETAR when agricultural practices were increasingly hidden as a response to the enforcement of conservation policies and laws (Fig. 2b). Since this resulted in insufficient food production, many small plots were reopened in 1999 (Fig. 2c). The receiving of state transfer payments and food packages and the sharing of plots and harvest in 2010 once again reduced the number of plots (Fig. 2d). Overall, there has been a reduction of agricultural activities in Bombas accompanied by a general increase in regenerating forest and forest (Fig. 3).

**Table 1:** Land cover in hectares and percent in the Bombas territory across four time periods (1962, 1990, 1999, and 2010)

Land use category	1962		1990		1999		2010	
	ha	%	ha	%	ha	%	ha	%
Agricultural activities	631.68	19	200.80	6	352.30	11	211.19	6
Forest in regeneration	341.72	11	416.38	13	356.95	11	473.39	15
Forest	2256.13	70	2612.36	81	2520.30	78	2544.95	79
TOTAL	3229.54	100	3229.54	100	3229.54	100	3229.54	100

The literature on shifting cultivation in tropical rainforests indicates that curtailment of agriculture can lead either to forest transition (Rudel 2012) or to agricultural intensification (Adams *et al.* 2013). In both cases, the supression of small-scale disturbances at lower levels, such as small fires, has been shown to result in lower biodiversity and structural complexity. Fire management that allows a mosaic of cultivated areas, secondary forest, and primary forest to develop has been shown to contribute to more diverse ecosystems (Russel 1997; Porro 2005; Pedroso *et al.* 2009; Beymer-Farris *et al.* 2012). This is because different forest ages support different plant species and interactions, and also permit different wildlife populations access to forest resources that vary in abundance across forest succession (Holling 1986; Rerkasem *et al.* 2009; Oudenhoven *et al.* 2011). The detected increase in regenerating forest and forest in Bombas suggests that the reduction of traditional shifting cultivation practices over the last decades has translated into an increase in total forested area and to forest transition as predicted by Rudel (2012). This is also supported by Fox *et al.* (2000) who argued that shifting cultivation is a temporary removal of trees, not of forest, properly speaking. Although the forest cover in Bombas has not been subject to any major change through time, the vegetation profile changed from a heterogeneous to a more homogenous forest.

### Social-Ecological Adaptations

**Fig. 4:**
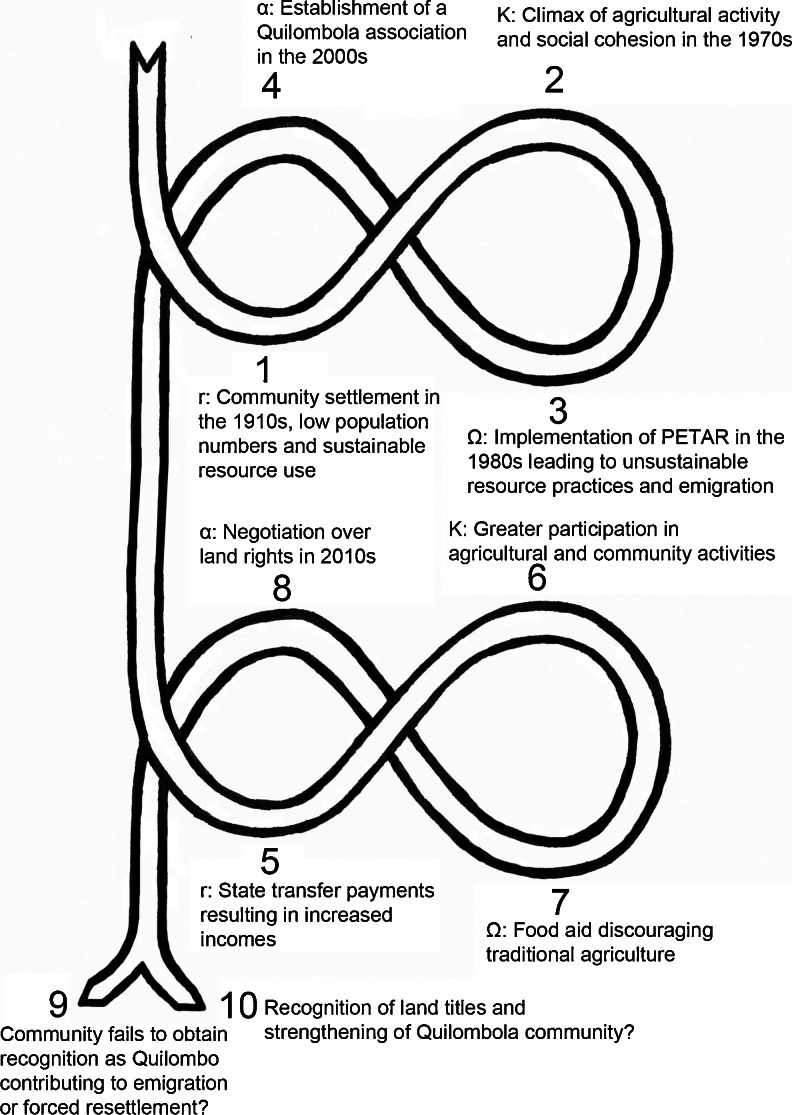
Two linked and consecutive adaptive cycles of the social-ecological system of Bombas depicting ecological, political, institutional, and social changes over time

The social-ecological system of Bombas has gone through two linked and consecutive adaptive cycles of ecological, political, institutional, and social change over the last century (Fig. 4). The system has gone through breakdown leading to social- political reorganization, but rather than a repetition of a single adaptive cycle, new institutions, ideas, and policies have provided inputs to the beginning of a new cycle which again may produce a third future cycle, linking the system not only to its past but also to its future (Fig. 4). This depiction differs from most resilience literature that portrays the adaptive cycle as a more closed system (e.g., Gunderson and Holling 2002; Widlock *et al.* 2012).

The entry point of Fig. 4 refers to indigenous peoples’ knowledge of local agro-ecological conditions, agricultural tools and practices, and characteristics of plants and animals that were passed on to the first settlers of Bombas, including through marriages, who in turn have passed it on to their children and grandchildren. Historical accounts highlight the sporadic use of the Bombas valley before the current settlement, so what is perceived as “virgin” forest might well have been utilized in historical times. At the time of community settlement in the 1910s, agricultural plots were left fallow for considerable periods, measures were taken to prevent the uncontrolled spread of fire, and cutting of trees on steep slopes and in riparian zones was avoided, demonstrating that inhabitants avoided unsustainable practices (1). Population numbers increased and agricultural practices intensified over the following 50 years (Fig. 2a). According to residents, population density, social networks, cultural practices, and agricultural activities reached a climax in the 1970s (2). They also described that the abundance of planted crops and fruits attracted large quantities of ungulates, rodents and birds, increasing the availability of game meat. The active use of shifting cultivation was believed to produce a patchier and more complex forest structure holding a wide range of habitat niches, possibly sustaining wild species diversity. Because of vigorous engagement in agricultural and other community activities, social cohesion was strong. The progression from (1) to (2) is associated with a slow increase in organization and connectedness over time and a gradual accumulation of natural, social, and human capital.

The implementation of PETAR in 1987 was experienced as a disturbance to this more or less stable social-ecological system (3). As a response to environmental restrictions and fear of being fined, unsuitable areas were cultivated in an attempt to conceal the activity, extraction of the threatened *juçara* palm increased, and crop production decreased resulting in fewer wild animals according to community members (Fig. 2b). Furthermore, increased skepticism among community members and distrust of outsiders, coupled with emigration, resulted in weakened social cohesion and loss of traditional practices and knowledge, and thereby loss of some crop varieties. On the other hand, this “release” stage or “creative destruction” gave room for innovation and renewal. The first adopted survival strategy was to cultivate a large number of smaller agricultural plots (Fig. 2c). The second response was to socially and politically reorganize the community in order to claim legal recognition as a *Quilombo*. The establishment of a *Quilombola* association in the 2000s was an attempt to legalize settlement and resource use and get access to social services and infrastructure (4).

The acquisition of key documents and receiving of state transfer payments resulted in increased income for some of the inhabitants, thus supporting fellow community members and paying for agricultural tasks (5). This initially contributed to increased participation in agricultural and community activities (6). Nevertheless, the distribution of food packages and cash transfer programs eventually discouraged traditional agricultural activities, increasing dependence on governmental assistance (7). As less food was produced, self-sufficiency diminished and the necessity for money increased (Fig. 2d). In 2013, it seemed that Bombas was poised for a new round of institutional renewal after entering negotiations over territory with the Forest Foundation to proceed with the *Quilombola* recognition process (8).

Based on the reports of Bombas residents and other engaged actors, some possible future scenarios in the back loop phase of the second adaptive cycle, from release to reorganization, may be delineated. One may be that the community fails to obtain official recognition as a *Quilombo* (9). This could result in forced resettlement of inhabitants, but more likely in a continual degradation of living conditions and emigration, ultimately leading to the abandonment of the Bombas settlement. Based on findings from the land cover analysis this would most likely result in regrowth of a forest that is more homogenous, leading to reduced ecological complexity and biological diversity, as indicated by Bombas residents and various scholars (e.g., Russel 1997; Pedroso Jr. *et al.* 2009; Oudenhoven *et al.* 2011; van Vliet *et al.* 2012; Robbins 2012; Beymer-Farris *et al.* 2012). For the community members, eviction from their historical territory could translate into their cultural identity being lost, as well as further loss of traditional practices and knowledge and degraded social relations. An alternative future scenario may be that the community is legally recognized as a *Quilombo*, obtaining a registered land title (10). Forest authorities could thereby move PETAR’s borders excluding the territory of Bombas, or alternatively re-classify the territory as a sustainable use area permitting human residence and activity. The social-ecological system could then create room for reorganization, renewal, and novelty. Access to improved infrastructure could enable transport of agricultural products to local markets, children to undertake further studies in nearby towns, the sick and pregnant to receive health assistance, and facilitate the initiation of small businesses, ecotourism, and market-oriented agricultural production as is the case in other adjacent *Quilombola* communities (Adams *et al.* 2013). Legal recognition of land rights could thus encourage engagement in subsistence activities and also improve inhabitants’ adaptive capacity in case of policy or economic changes.

## Conclusion

Over the last century, Bombas has experienced two main cycles of change in social and ecological terms. By combining the adaptive cycle from resilience literature and political ecology, I have shown that the interaction among various development, environmental, and social policies and interventions has affected Bombas inhabitants’ land use with cumulative effects on their livelihoods and the ecology of the Atlantic Forest. Development initiatives in the 1930s-1970s attracted people to Bombas who provided additional labor, but concomitantly led to more opportunities being established outside the territory and to emigration of inhabitants, particularly younger ones, in search of a better life. Environmental policies prohibiting human occupation and resource use later led to further emigration and subsequent reduced engagement in subsistence activities. Social policies in the 2000s resulted in higher income allowing inhabitants to buy products otherwise planted or processed in the community or to substitute traditional products with items provided by government food packages. The combined effects of these processes resulted in a reduction of shifting cultivation practices in Bombas and an increase in forest cover.

Based on informants’ accounts and land cover maps, I argue that Bombas residents have played a significant role in shaping and maintaining the Atlantic Forest by past and present resource management practices. The mosaic of small agricultural plots, areas in regeneration, and forest areas promote niche diversity with favorable conditions for the diversification of wild and cultivated plant and animal communities. The empirically evident regrowth of Atlantic Forest followed by a decreased engagement in agricultural activities suggests that there have been no serious long-term negative impacts on the forest cover and that Bombas inhabitants have not exceeded the capacity of the soil to sustain both agricultural production and conservation. This is contrary to the dominant perception that traditional small-scale livelihoods are unproductive, destructive, and the cause of environmental degradation, a depiction that is utilized to legitimize the establishment of strictly protected areas (Pedroso *et al.* 2009; Robbins 2012; Beymer-Farris 2013). Oudenhoven *et al.* (2011) highlight that landscapes that have co-evolved with human activities often depend on their continuation to maintain the presence of certain species and ecosystem services. Based on this reasoning, biodiversity conservation could potentially benefit more from the inclusion and empowerment of Bombas residents and encouragement of their knowledge, practices, and culture characterizing the traditional agricultural system, than from their exclusion. I therefore conclude that legalization of settlement and subsistence activities are important not only for livelihood security and social cohesion of local inhabitants, but possibly also for biodiversity conservation. This should be taken into account in future negotiations and planning processes between the Bombas *Quilombola* association, the Forest Foundation, and the Land Institute of São Paulo concerning land rights to the territory of Bombas and natural resource management. If Bombas is recognized as a *Quilombo*, its residents will be in a favorable position to negotiate their future with the State for the first time in their history.

## Footnotes

1 The word *Quilombo*, or maroon, refers to a settlement of descendants of either run-away slaves, slaves who bought their freedom, or freed slaves who received land by donation or through heritage, or who occupied abandoned or unoccupied government lands (Schmitt *et al.* 2002). *Quilombola* is the adjective of Quilombo and can refer to a resident, a community, an association, a tradition, etc.

2 Brazil officially abolished slavery in 1888.

3 The establishment of a number of sustainable use areas since the mid-1980s, permitting human settlement and low impact resource use, shows a positive trend towards more socially and environmentally just conservation approaches in Brazil.

4 The aerial photo was obtained from the Department of Geography at the University of São Paulo while the Landsat satellite images from 1990 and 1999 and the SPOT satellite image from 2010 were obtained from the socio-environmental NGO Instituto Socioambiental (ISA) in São Paulo.

5 Bombas inhabitants differentiate among various mutual-help organizations; *puxirão/mutirão*, *reunida*, *troca de dia*, and *camarada*. *Mutirão*, a newer word for *puxirão*, refers to a large-scale mutual-help organization, comprising group activities for clearing a forest patch, planting, weeding, and harvesting. *Reunida* is collective work for a collective end, like opening a new trail, or individual end like construction of a house. *Troca de dia* is when one person helps another one day in exchange for help another day. *Camarada* is when one person pays someone to help.

6 Carlos Lamarca was one of the leaders of the armed opposition to the military dictatorship in Brazil.
